# Successful Premaxillary Reconstruction and Oronasal Fistula Closure in a Patient With VATER Syndrome With Bilateral Cleft and Missing Premaxilla

**DOI:** 10.1177/10556656251408193

**Published:** 2025-12-29

**Authors:** Jayson Workman, Antonio Atte, Patrick Wong, Fabio Ritto

**Affiliations:** 1 Oral and Maxillofacial Surgery, 12334 UT Southwestern Medical Center, Dallas, TX, USA; 2 Oral and Maxillofacial Surgery, 6186 University of Oklahoma Health Science Center, Oklahoma City, OK, USA

**Keywords:** bone grafting, bone regeneration, cleft lip and palate, craniofacial surgery, dental arch, maxilla

## Abstract

VATER syndrome is a non-random association of birth defects affecting multiple organ systems, with orofacial clefts uncommonly reported. This study presents a unique case of a patient with VATER syndrome with a bilateral cleft lip and palate who underwent the rare surgical removal of the premaxilla during cleft lip repair, resulting in a large maxillary defect and oronasal fistula. This report highlights the successful use of non-vascularized bone graft in this setting, with 13-month follow-up demonstrating bone consolidation and fistula closure. The case adds to the evidence supporting non-vascularized bone graft for extensive maxillary defects, even with oronasal fistula.

## Introduction

VATER syndrome, also known as VACTERL syndrome, is a syndrome with no identified genetic or chromosomal issue and is considered a diagnosis of exclusion by >3 of core malformations. The reported prevalence of clefts are not a core feature of the VATER group, however, VACTERL anomalies present in the same development window as clefts—4-10 weeks for clefting and 3-6 weeks for VACTERL suggesting a shared disruption in mesoderm development. In a study of VACTERL patients looking at International Clearinghouse registries, only 3% to 5% of patients presented with cleft, highlighting the rarity of this presentation.^1^

Premaxillary reconstruction can be a particularly difficult challenge for the cleft surgeon. Many different reasons can lead to a missing premaxillary segment including congenital absence, discarding during early cleft procedures, or necrosis after surgery. The existing literature regarding the subject has mixed treatment modalities between autogenous bone grafting versus using a vascularized bone graft reconstruction of the missing premaxilla.^2–5^ Since no standardization of treatment exists, this leads the surgeon to make decisions for treatment based on the few available case reports. The decision between vascularized and non-vascularized grafts has a significant impact on patient morbidity and must be made with consideration of these factors and their individual success rate.^6–8^ Here we present a case of successful premaxillary reconstruction using a non-vascularized anterior iliac crest block graft.

## Patient Presentation

**Figure 1.**
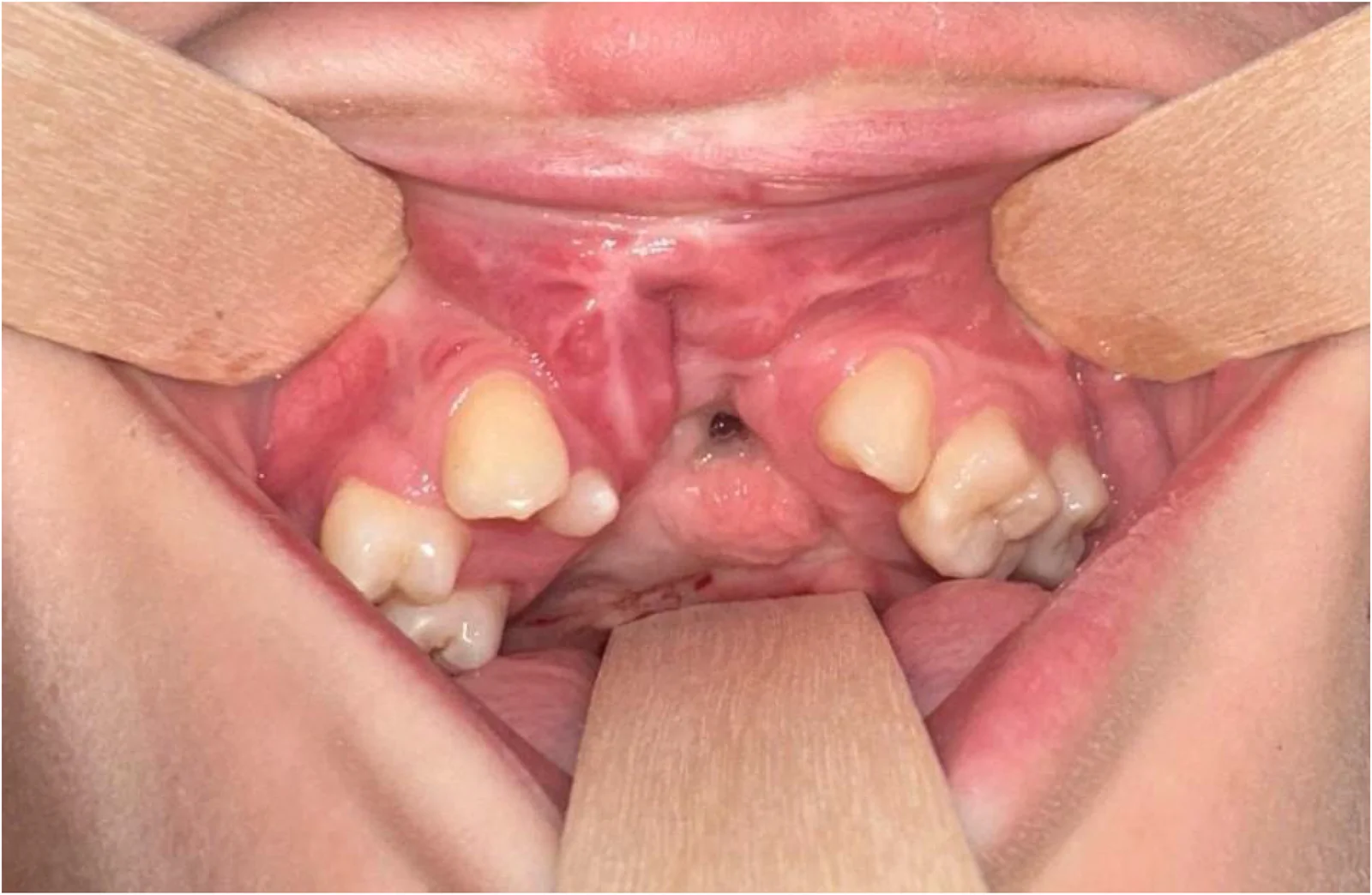
Intraoral photograph demonstrating the large missing premaxillary segment and oronasal fistula.

**Figure 2.**
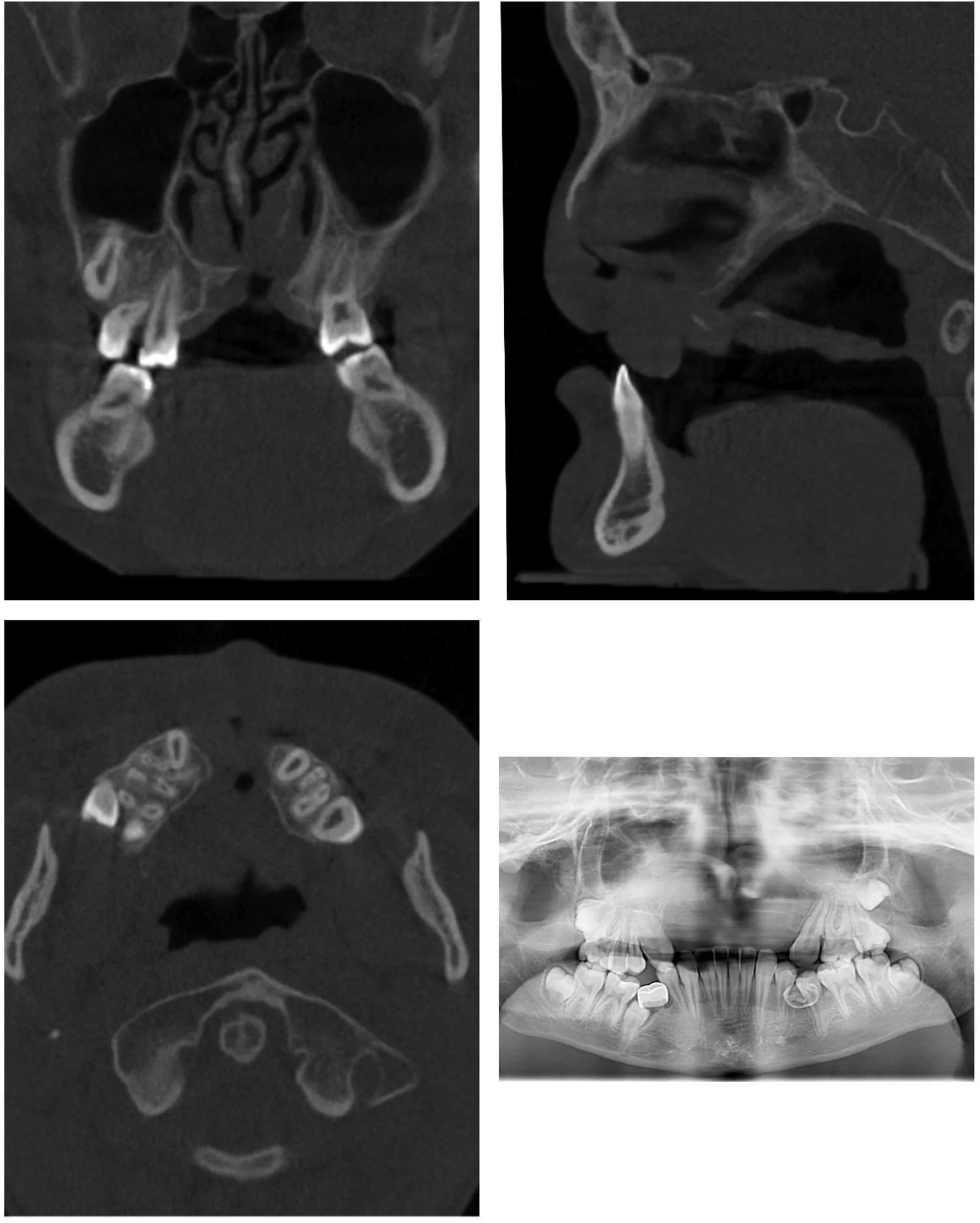
CT scan with single cuts from coronal (upper left), sagittal (upper right), axial (lower left), and panoramic views (lower right). Scans taken pre-procedure demonstrating missing premaxillary segment.

The patient was a 12-year-old male with a diagnosis of VATER syndrome and a complete bilateral cleft lip and palate. The patient had his premaxilla surgically removed by a previous surgeon during his primary lip repair. At presentation, the patient had a large oronasal fistula intraorally (Figures 1 and 2) due to his missing premaxilla with reports of significant liquid passage into his nose during eating and drinking. The patient was in mixed dentition, did not have any orthodontic preparation, had missing anterior teeth #7, 8, 9, and 10, and had a supernumerary tooth #56 adjacent to the defect. The patient was planned for surgical reconstruction with anterior iliac crest bone graft, extraction of supernumerary tooth #56, and oronasal fistula closure. The patient presented with bilateral posterior crossbite. This was not addressed preoperatively due to concerns that maxillary expansion would widen the cleft gap leading to larger and more difficult bone graft. After bone graft and skeletal maturity, the patient will be planned for maxillary advancement to address his maxillary hypoplasia and negative overjet.

### Surgical Technique

The anterior iliac crest was selected due to low donor site morbidity, accessibility, and adequate bone volume for harvest.^8^ A 2 cm wide cortical block was harvested from the medial aspect of the left iliac crest using a reciprocating saw and osteotome. After harvesting the cortical block graft, cancellous bone was harvested from the iliac crest using bone curettes. After procurement of the graft, the site was irrigated, and the hip surgical site was then closed with appropriate multilayer closure.

Vertical incisions were made medial to the maxillary canines and were extended to the anterior aspect of the patient's midline alveolar fistula bilaterally. Subperiosteal dissection was done to expose the lateral segments of the maxilla and the bony edges of the cleft defect were identified up to the piriform rim.

**Figure 3.**
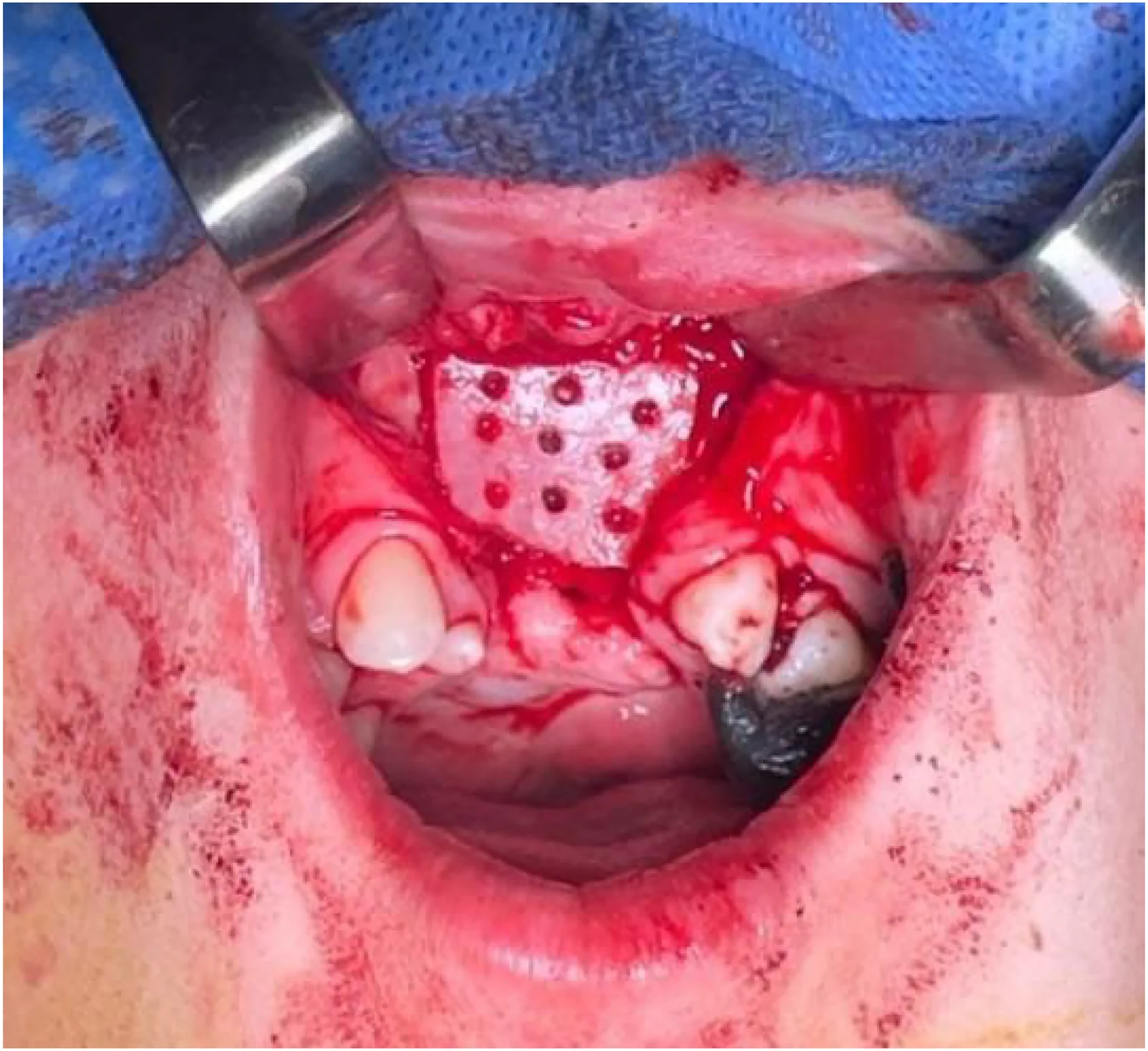
Inset of block harvested from the ileum with perforations with Rh-BMP2-soaked collagen sponge on nasal mucosal layer.

The nasal mucosa was separated from the buccal and palatal mucosa. The nasal mucosa was then closed to form a watertight seal. The cortical block was then shaped and then perforations made through the block to aid in vascular ingrowth and integration. A Rh-BMP2 (Infuse-Medtronic) soaked collagen sponge was then placed against the closed nasal mucosal layer as well as the closed palatal mucosa. The cortical block was gently inserted into the cleft defect with a mallet to achieve primary stability without excessive force (Figure 3). Once adequately secured, the cancellous bone harvested from the ilium was packed into any defects and an additional Rh-BMP2 covered the anterior and inferior aspects of the graft. Rh-BMP2 was used to promote osteogenesis and graft incorporation. No complications noted and the patient was turned over to anesthesia for extubating and transportation to the PACU.

**Figure 4.**
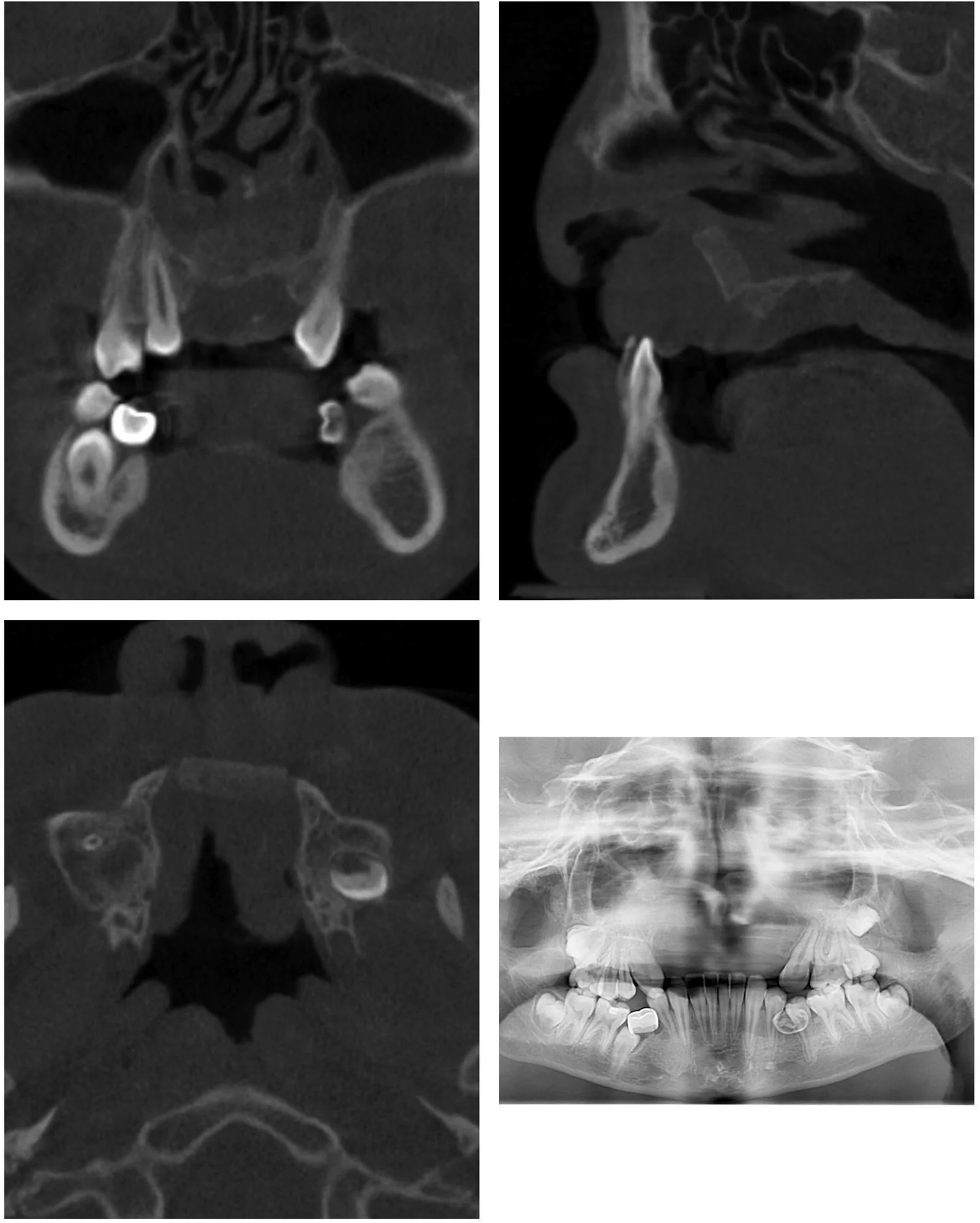
CT scan with single cuts from coronal (upper left), sagittal (upper right), axial (lower left), and panoramic views (lower right). Scans taken at 1 week post procedure demonstrating evidence of cortical block placement at premaxilla.

**Figure 5.**
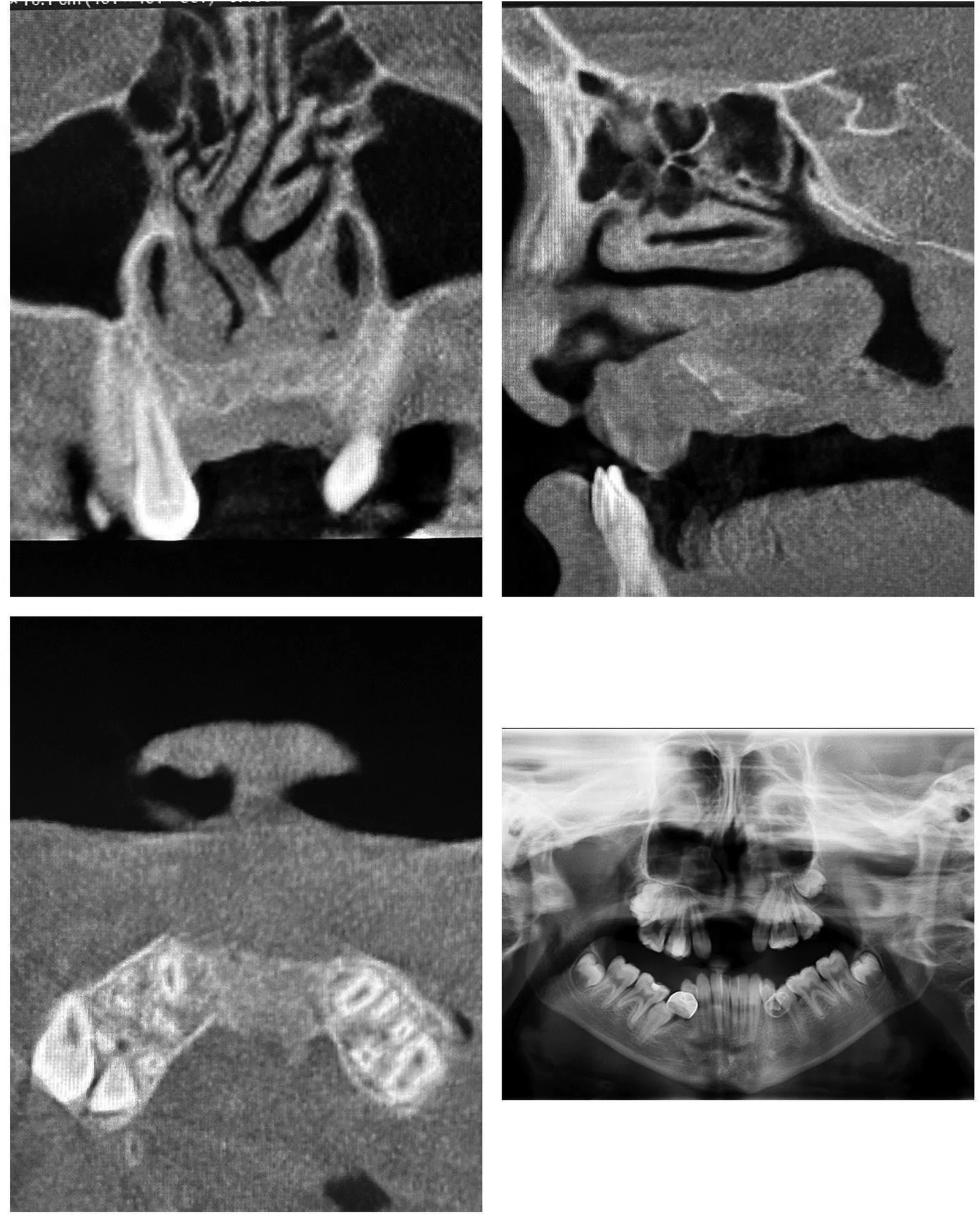
CT scan with single cuts from coronal (upper left), sagittal (upper right), axial (lower left), and panoramic views (lower right). Scans taken at 3 months post procedure demonstrating evidence of bony fill of premaxillary segment.

**Figure 6.**
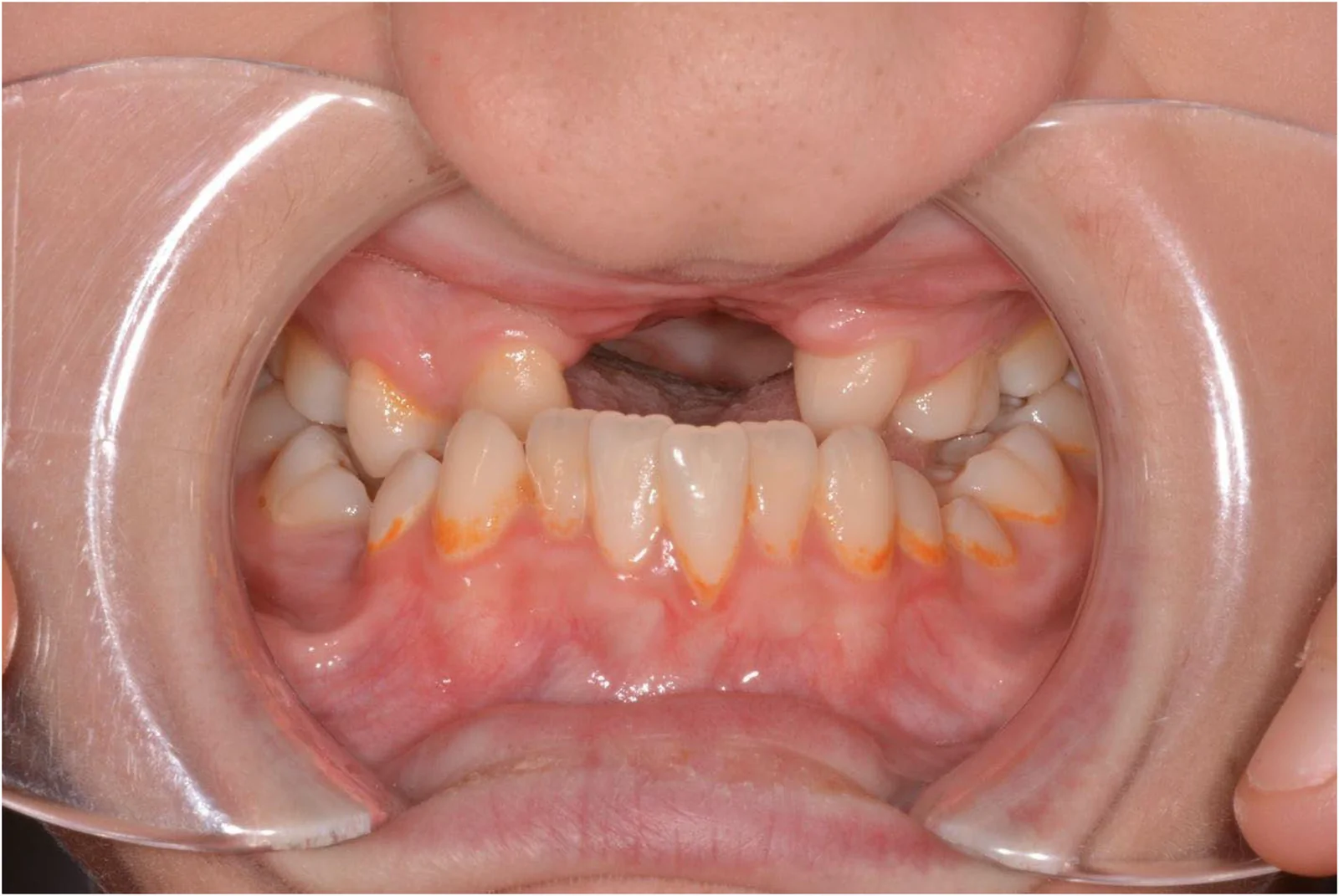
Intraoral photograph taken 3 months post-op. Bilateral class III malocclusion is visualized.

**Figure 7.**
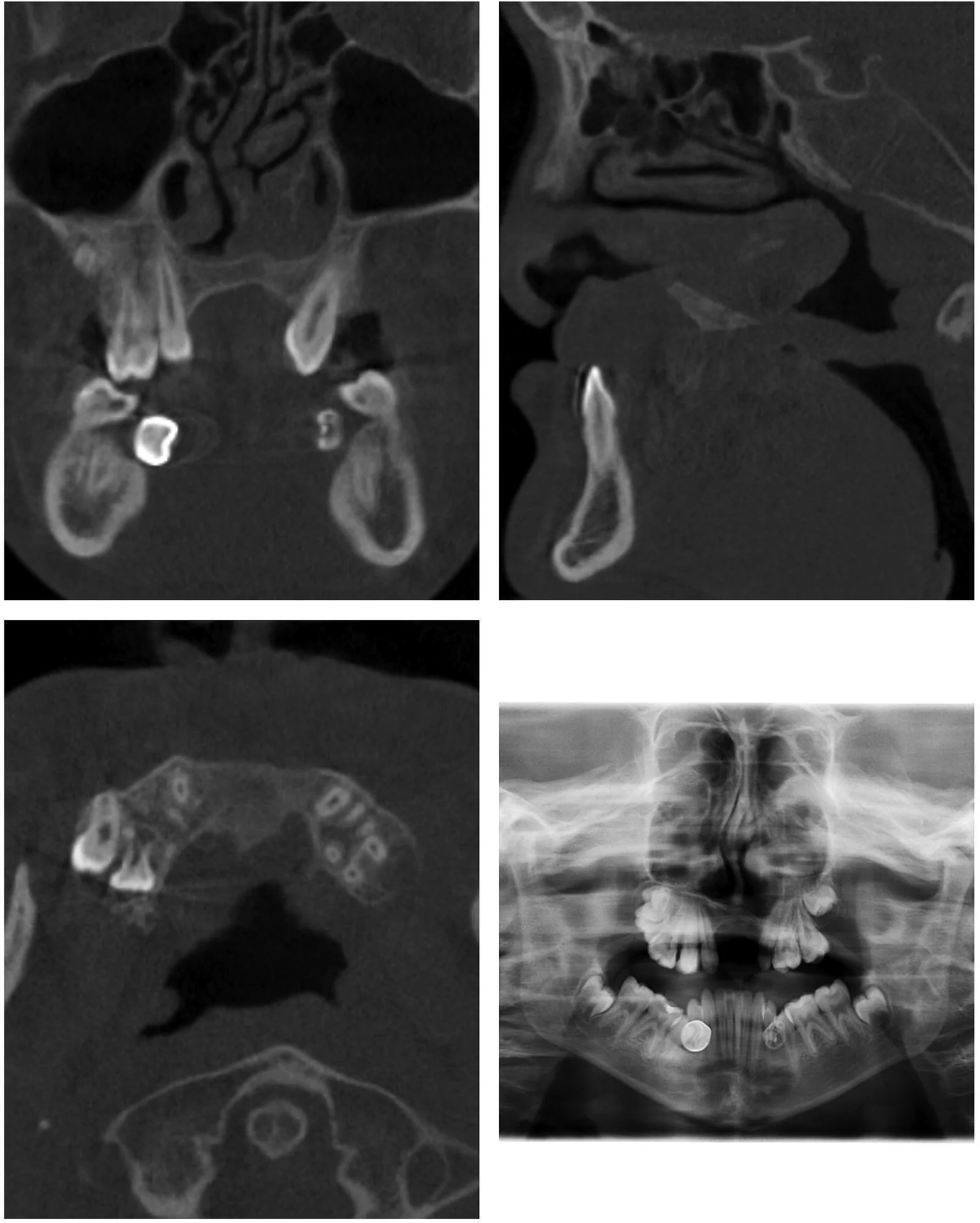
CT scan with single cuts from coronal (upper left), sagittal (upper right), axial (lower left), and panoramic views (lower right). Scans taken at 13 months post procedure demonstrating evidence of continued bony fill of premaxillary segment.

The patient thereafter had an unremarkable hospital stay and was discharged on post-operative day 1. Post-operative instructions were given for 6 weeks of soft diet and 1 week of post-operative antibiotics with Augmentin. At 1 week and 3-months post-op, the patient had photographic and radiographic evaluation (Figures 4-6). At 13-month post-op, the patient had additional radiographs taken (Figure 7) all demonstrating stable bony fill after the operation. Final measurements of bone graft from Figure 7 at 13 months were 4.95 mm×13.05 mm at the most central portion of the graft.

## Literature Review

A comprehensive retrospective review was conducted utilizing PubMed and Google Scholar to identify scientific articles detailing cases of patients with bilateral cleft lip and palate and a missing premaxilla. The search employed keywords such as “bilateral cleft lip and palate,” “missing premaxilla.” Inclusion criteria were restricted to studies published in English that described patients with bilateral cleft lip and palate and a missing premaxilla. The search strategy yielded 7 studies. Each one highlights different reconstructive strategies and outcomes.

Abreu et al. (2021) presented a patient with bilateral cleft lip and palate and missing premaxilla.^9^ The patient underwent orthodontic treatment in preparation for orthognathic surgery and interim esthetic management with a maxillary partial denture during that time. The patient had a 2-piece maxillary advancement with iliac crest bone graft bony union without any use of rh-BMP2. Implant restoration was desired but unable to be performed due to lack of alveolar bone. A fixed partial denture was then fabricated using bilateral maxillary canine and first premolars are abutment teeth and teeth #7-10 being pontic teeth.

Park et al. (2022) reported on a patient with bilateral cleft lip and palate and a missing premaxilla.^5^ The patient had lip repair, palate repair, rhinoplasty, pharyngeal flap, initial orthodontics, and 2 attempts at alveolar bone grafting prior to presenting at their institution. At their center, the patient had orthodontic treatment to prepare for maxillary distraction osteogenesis. After surgery, orthodontic treatment was finalized. Final esthetic and prosthodontic rehabilitation were completed with a fixed partial denture. This case underscores the value of multidisciplinary approaches with specialists including orthodontics, prosthodontics, and surgery.

Altuntaş and Aydin (2022) described a patient with bilateral cleft lip and palate, a rudimentary premaxilla, and large fistula following failed bone grafting.^2^ The surgical team decided to use a free vascularized iliac bone flap for reconstruction and then performed dental implant placement. This case demonstrated the viability of free tissue transfer as a rescue method in difficult outcomes and a method to have sufficient bone for implant placement.

Fukuda et al. (2003) presented on a patient with bilateral cleft lip and palate and missing premaxilla.^4^ Reconstruction was performed using iliac crest bone grafting, without the use of rh-BMP2, for their reconstruction. This procedure provided sufficient bone to place endosteal implants and placement of a fixed prosthesis. This allowed for the functional and esthetic outcome without the need for free tissue transfer.

Ortiz Monasterio et al. (2009) reported on a series of 7 patients with missing premaxillae. The reconstruction approach for premaxillary reconstruction combined LeFort I surgery, mucosal grafting, and staged fibula free flap surgery.^10^ This comprehensive strategy enabled osseointegrated implant placement with all patients.

Dibbs et al. (2021) described a 19-year-old patient with bilateral cleft lip and palate and a missing premaxilla, who had previously undergone lip repair, multiple revisions, premaxillary resection, rhinoplasty, turndown flaps for oronasal fistula closure, iliac crest bone grafting, and Abbe flap.^11^ At presentation, the patient had a persistent oronasal fistula and a large premaxillary defect. Management involved a staged approach: initial closure of the oronasal fistula with a radial forearm free flap, followed by reconstruction of the anterior maxilla using a fibula free flap with integrated dental implants and a 2-piece LeFort I advancement. This case highlights the complexity of secondary reconstruction in cleft patients with extensive maxillary defects and demonstrates the utility of free tissue transfer for both soft tissue closure and bony reconstruction, particularly when previous interventions have failed.

Torroni et al. (2007) reported their experience using fibula free flaps to reconstruct the premaxillary region in patients with acquired maxillary defects—particularly those involving the anterior maxilla and premaxilla—where traditional bone grafting methods were inadequate.^12^ The study reviewed a series of patients who underwent premaxillary reconstruction with vascularized fibula free flaps. The authors described indications, surgical planning, flap design, and outcomes. Virtual planning was not yet routine in 2007, so they emphasized meticulous intraoperative adaptation of the fibula segment to the premaxillary contour. The flap's vascular pedicle length and robust bone stock allowed rigid fixation to the remaining maxilla and potential for future dental rehabilitation.

## Discussion

The surgical management of patients with a history of cleft and missing premaxilla represents a rare and challenging clinical scenario. This is reported infrequently and there is no consensus on optimal reconstructive approach.^2,4,5,9,10^ Without a native premaxilla, the reconstructive surgeon must first recreate the anterior maxillary foundation, typically through autogenous bone grafts or free tissue transfer. The lack of adequate soft tissue, scarring from multiple prior surgeries, and poor vascularity further complicate outcomes. Clinical features common to these patients include midfacial retrusion, collapsed alveolar arch, and collapsed nasal projection. Without anterior bony support, the columella and nasal tip collapse, compromising nasal projection and lip reconstruction. This case adds to the limited literature by demonstrating that non-vascularized anterior iliac crest block grafting, with adjunctive rh-BMP2, can lead to stable and functional results for premaxillary reconstruction in the setting of a missing premaxilla.

Previous literature has reported success using vascularized bone flaps for secondary maxillary reconstruction. Vascularized free flaps are often seen as gold standard in large reconstruction cases because of their high success rate and predictability.^13^ Both fibula and deep-circumflex iliac artery free flaps have been used in case reports for premaxillary reconstruction^2,10^ which carry risks of persistent issues with pain, sensation, gait, walking, cosmetic appearance, and physical activity.^6,7^ The operations are technically complex. Additionally, the hospital stay is longer for individuals with free tissue transfers compared to non-vascularized grafts.^14^

In contrast, non-vascularized iliac crest bone grafts represent a less invasive and time-efficient alternative. The iliac crest provides corticocancellous bone with excellent osteogenic potential and contour adaptability for anterior maxillary reconstruction. Morbidity is generally low and includes transient pain, hematoma, sensory disturbance, and gait alteration, though risk increases with larger graft harvests.^8^ In our case, a single overnight hospital stay was sufficient prior to discharge. While non-vascularized grafts carry a theoretical risk of resorption or failure in poorly vascularized fields. Careful soft-tissue handling, adequate flap coverage, and the adjunctive use of osteoinductive materials such as rh-BMP2 can mitigate these risks and enhance long-term stability.

In the literature, traditionally alveolar cleft graft cases in the pediatric population, one night was sufficient to discharge a patient from the hospital, highlighting the reduced perioperative burden. In patients with vascularized tissue transfer, increased monitoring of flap vitality and donor site recovery is needed typically for at least a week.^14^ However, free flaps often provide greater bone volume, which can facilitate dental implant placement.^2,10,15^ This may be an issue in our case where good bone volume is present but may be insufficient for dental implant placement. All these factors, including the desired function and esthetic outcomes, must be taken into consideration in the final treatment decision. As evidence and methods improve, the implementation of non-vascularized grafts and other less invasive techniques may offer significant benefit, with decreased hospital stay and morbidity, when cases are appropriately selected.
